# Optical Coherence Tomography Angiography in Patients with Systemic Lupus Erythematodes

**DOI:** 10.1055/a-2792-0840

**Published:** 2026-04-20

**Authors:** Maximilian Robert Justus Wiest, Timothy Hamann, Katrin Fasler, Teresa Trunfio, Mario Damiano Toro, Sandrine Zweifel

**Affiliations:** 1 Ophthalmology, University Hospital Zurich, Switzerland; 2 Eye Clinic, Department of Public Health, University of Naples Federico II, Napoli, Italy; 3 Chair and Department of General and Paediatric Ophthalmology, Medical University of Lublin, Poland

**Keywords:** retina, uveitis, intraocular inflammation, Retina, Uveitis, systemischer Lupus erythematodes

## Abstract

**Purpose** Systemic lupus erythematodes (SLE) is a connective tissue disorder with multisystem involvement that leads to an immune complex-mediated angiopathy with deposition in the endothelial cells of small blood vessels. The purpose of this study was to analyse the retinal vasculature, using optical coherence tomography angiography (OCTA) in patients with SLE.

**Methods** This is a single-institution cross-sectional study. Macular OCTA images (6 × 6 and 3 × 3 mm) were acquired in healthy controls (HC) and patients with SLE. En face images of the retinal vasculature were generated from the superficial and deep capillary plexus (SCP/DCP). The vessel density (VD) and vessel length density (VLD) were measured by previously published methods. VD was assessed as the ratio of the retinal area occupied by vessels. VLD was measured as the total length of the skeletonised vessels using 1-pixel center line extraction of the blood vessels. Quantitative analysis of the VD, VLD, and the foveal avascular zone FAZ was performed.

**Results** 25 patients (49 eyes) with clinically established SLE and 80 eyes of 66 healthy controls (HC) were included. Among the patients with SLE, the mean age was 41.47 (± 9.27 SD) years with 92% female patients and in the healthy individuals 37.1 years (± 14.33 SD) with 56% female patients. The mean VD of the SCP in the SLE-group was 0.34 ± 0.02, the VLD of SCP was 17.08 ± 1.58 mm ^−1^ , the VD of DCP was 0.28 ± 0.03 and the VLD of DCP was 14.21 ± 1.76 mm ^−1^ in the 3 × 3 mm scans. In the control group, the mean VD of SCP was 0.36 ± 0.03, the VLD of SCP was 17.50 ± 1.97 mm ^−1^ , the VD of DCP was 0.31 ± 0.04 and the VLD of DCP was 15.86 ± 4.19 mm ^−1^ . There was a statistically significant reduction in both VD of SCP and DCP and of the VLD of the SCP in SLE group (p < 0.05) compared to controls.

**Conclusions** Swept-source OCTA of the microcirculation in eyes of patients with SLE can be used to quantitatively demonstrate a reduction in VD in SCP and DCP and VLD in SCP compared to healthy eyes. The reduction of the capillary network in the superficial and deep capillary plexus indicates an association with SLE disease status and future longitudinal studies may use these metrics to evaluate changes over time and its correlation to disease severity. Further studies are warranted to evaluate the relevance of these microvascular alterations in the setting of SLE and its correlation to disease severity.

## Zusammenfassung

**Zweck** Systemischer Lupus erythematodes (SLE) ist eine Bindegewebserkrankung mit Multisystembeteiligung, die zu einer immunkomplexvermittelten Angiopathie mit Ablagerungen in den Endothelzellen kleiner Blutgefäße führt. Ziel dieser Studie war es, die Netzhautgefäße bei Patienten mit SLE mithilfe der optischen Kohärenztomografie-Angiografie (OCTA) zu analysieren.

**Methoden** Makuläre OCTA-Bilder wurden bei gesunden Kontrollpersonen (HC) und Patienten mit SLE aufgenommen. En-face-Bilder der Netzhautgefäße wurden aus dem superfiziellen und tiefen Kapillarplexus (SCP/DCP) erzeugt. Die Gefäßdichte (VD) und die Gefäßlängendichte (VLD) wurden nach zuvor veröffentlichten Methoden gemessen. Die VD wurde als Verhältnis der von Gefäßen eingenommenen Netzhautfläche bestimmt. Die VLD wurde als die Gesamtlänge der skeletonisierten Gefäße anhand einer 1-Pixel-Mittellinienextraktion der Blutgefäße gemessen. Eine quantitative Analyse der VD und der VLD wurde durchgeführt.

**Ergebnisse** 25 Patienten (49 Augen) mit klinisch gesichertem SLE und 80 Augen von 66 gesunden Kontrollpersonen (HC) wurden eingeschlossen. Das Durchschnittsalter der SLE-Patienten betrug 41,47 (± 9,27 SD) Jahre, mit einem Frauenanteil von 92%. Bei den gesunden Probanden betrug das Durchschnittsalter 37,1 (± 14,33 SD) Jahre, mit einem Frauenanteil von 56%. In den 3 × 3 mm-Scans betrug die mittlere VD des SCP in der SLE-Gruppe 0,34 ± 0,02, die VLD des SCP 17,08 ± 1,58 mm ^−1^ , die VD des DCP 0,28 ± 0,03 und die VLD des DCP 14,21 ± 1,76 mm ^−1^ . In der Kontrollgruppe betrug die mittlere VD des SCP 0,36 ± 0,03, die VLD des SCP 17,50 ± 1,97 mm ^−1^ , die VD des DCP 0,31 ± 0,04 und die VLD des DCP 15,86 ± 4,19 mm ^−1^ . Es zeigte sich eine statistisch signifikante Reduktion sowohl der VD des SCP und DCP als auch der VLD des SCP in der SLE-Gruppe im Vergleich zur Kontrollgruppe (p < 0,05).

**Schlussfolgerungen** Die Swept-Source-OCTA der Mikrozirkulation in den Augen von SLE-Patienten kann quantitativ eine Verringerung der VD im SCP und DCP sowie der VLD im SCP im Vergleich zu gesunden Augen nachweisen. Die Reduktion des Kapillarnetzwerks im superfiziellen und tiefen Kapillarplexus könnte relevant sein. Zukünftige longitudinale Studien können diese Messgrößen verwenden, um Veränderungen im Zeitverlauf und deren Zusammenhang mit dem Krankheitsverlauf zu bewerten. Weitere Studien sind erforderlich, um die Relevanz dieser mikro-vaskulären Veränderungen im Kontext von SLE, deren Zusammenhang mit der Krankheitsaktivität zu untersuchen.

## Introduction

Systemic lupus erythematodes (SLE) is a chronic multisystem autoimmune disease frequently involving the eyes, potentially leading to inflammation in any ocular structure 1 , 2 . Continuous deposition of immune complexes in the basement membrane of blood vessels impairs diffusion of oxygen and other molecules, which can cause microangiopathy and especially vascular occlusions 1 , 2 . The American College of Rheumatology has defined 11 features at least 4 of which need to be present for establishing the diagnosis if SLE. The revised criteria include: malar rash, discoid rash, skin photosensitivity, oral ulcers, nonerosive arthritis, serositis, renal involvement, neurological disorder, hematologic disorder, immunologic disorder, and positive antinuclear antibodies 3 , 4 . The serial or simultaneous presence of 4 of these 11 criteria allows the confirmation of the diagnosis of SLE with high sensitivity and specificity. Currently, there are no objective ophthalmological criteria involved in establishing the diagnosis of SLE, even though up to one third of patients present themselves with ocular involvement 5 .

Amongst the clinical ocular manifestation of SLE are retinal vascular alterations. The increased risk for thrombotic events associated with antiphospholipid syndrome can lead to severe venous occlusions and resulting damage to the retinal vascular bed 2 , 6 . Additionally, autoimmune inflammation, mediated through deposition of immune complex material can lead to microvascular occlusions, increasing the risk for nonperfused retinal areas 7 . Most authors consider visible retinal damage as marker for a high disease activity, while recent studies have suggested that patients with less severe or better controlled disease might display subclinical functional retinal impairment 8 . A method able to detect this subclinical retinal damage might therefore facilitate the early diagnosis of SLE.

The retinal involvement has been assessed using optical coherence tomography, perimetry and rarely fluorescein-angiography, all of which are unable to detect the smallest microangiopathic changes 9 . Optical coherence tomography angiography (OCTA) is a non-invasive imaging technique capable of analyzing both the structural and angiographic depth-resolved information of the retina without application of intravenous dye 10 . In this study, we try to detect microvascular changes in patients suffering from SLE using OCTA, by comparing retinal scans of a SLE cohort with those of healthy patients.

## Materials and Methods

This is a single-institution, retrospective cross-section study. The institutional review board approved this study (BASEC-No.: 2019-02043). Imaging data acquired from patients suffering from SLE or age-matched healthy controls (HC) were included respectively.

Inclusion criteria were: Confirmed SLE diagnosis (not for healthy controls), 18 years of age or older. Exclusion criteria were: no informed consent given. Presence of retinal or ocular disease. High myopia of − 6 D or more. Relevant ocular media opacity impairing imaging quality (signal strength of 7/10 or less during OCTA acquisition). Imaging artifacts significantly impairing quantitative OCTA measurements such as defocus or motion artifacts 11 .

All patients underwent an ophthalmological examination with best corrected visual acuity (BCVA), intraocular pressure (IOP) measurement, slit lamp examination, spectral-domain optical coherence tomography (SD-OCT) and swept-source OCTA.

Swept-source OCTA imaging was acquired using a PLEX Elite 9000 device, software version 2.0.1.47652 (Carl Zeiss Meditec Inc., Dublin, CA, USA) device. 3 × 3 mm scans of the central retina were acquired and segmented into superficial capillary plexus and deep capillary plexus segmentation by the manufacturerʼs software. The vessel density and the vessel length density were then calculated using previously published methods 10 . The vessel density (VD) represents the fraction of pixels with detectable blood flow signal compared to the total amount of pixels in the scan area. The vessel length density (VLD) represents the total length of vasculature in mm per mm ^2^ (mm ^−1^ ) in the scan area.

### Statistical analysis

SPSS software (version 26, IBM Corporation, Armonk, NY, USA) was utilized for the statistical analysis. Baseline data were primarily displayer using descriptive statistics.

Then, the acquired OCTA values (VD and VLD) were analyzed to detect differences in between the SLE and HC group. Age and sex were controlled for by introducing them as cofactors in a type III fixed effect Test. VD and VLD in the SCP and the DCP were analyzed. Results were descriptively displayed in a table.

## Results

Forty-nine eyes of 25 patients suffering from SLE and 80 eyes from 66 healthy probands were included in this study. Baseline demographic data and additional data regarding disease severity score and Plaquenil therapy can be appreciated in Table 1 .

**Table:** **Table 1** Baseline Data.

	SLE Patients (n = 25)	Healthy Cohort (n = 66)
Male/Female	2/23	29/37
Mean age (years)	41.47 ± 9.27	37.1 ± 14.33
Right	24	38
Left	25	42
Disease Severity Score		
Mild	19	–
Moderate	5	–
Severe	1	–
Plaquenil-therapy		
No	4	–
Yes	21	–

For the SLE cohort, the mean VD and VLD in SCP were 0.34 ± 0.02 and 17.08 ± 1.58 mm ^−1^ respectively. In the DCP, VD and VLD were 0.28 ± 0.03 and 14.21 ± 1.76 mm ^−1^ . In the HC, VD and VLD in SCP and DCP were 0.36 ± 0.03, 17.50 ± 1.97 mm ^−1^ , 0.31 ± 0.04 and 15.86 ± 4.19 mm ^−1^ respectively. A statistically significant difference was detected in VD (p = 0.001) and VLD (p = 0.010) of SCP and in the VD (p = 0.004) of the DCP. These results can be appreciated in Table 2 .

**Table:** **Table 2** Statistical Analysis Results.

		SLE (n = 49)	Healthy (n = 80)	p value
SCP	VD	0.34 ± 0.02	0.36 ± 0.03	**0.001**
	VLD	17.08 ± 1.58	17.50 ± 1.97	**0.010**
DCP	VD	0.28 ± 0.03	0.31 ± 0.04	**0.004**
	VLD	14.21 ± 1.76	15.86 ± 4.19	0.065
Abbreviations: SLE: Systemic Lupus Erythematodes; SCP: superficial capillary plexus; DCP: deep capillary plexus; VD: vessel density; VLD: vessel length density				

## Discussion

In this retrospective analysis, statistically significant differences in VD and VLD in the SCP and in VD in the DCP in between a SLE and a healthy cohort were detected, with VD and VLD being lower in the SLE cohort.

SLE is an autoimmune disorder known to affect and vascular tissue through inflammatory processes and thrombotic events 1 , 6 . Over time, these changes can lead to profound changes and damages in the affected tissues, which, in case of the retina might lead to significant ischemic damage with resulting loss of visual acuity, retinal detachment or extended neovascular disease 7 . As with most chronic autoimmune disorders, early diagnosis and treatment can lead to better outcomes with less burden of disease and increased quality of life 12 . The ability to detect subclinical changes in eyes otherwise classified as healthy might support already established diagnostic algorithms and disease activity scores in identifying and monitoring patients with an increased risk of progressive disease 4 .

In this study, a difference in quantitative measurements of the SCP and DCP of the perifoveal retinal vasculature was detected. These findings suggests that early signs of microangiopathic changes, which might be precursors to SLE retinopathy, are detectable by OCTA imaging, a finding which other authors also report 13 , 14 . The pathway leading to a reduction of VD in SCP and DCP and VLD in SCP remains unclear, but the fact that both VD and VLD seem to be affected in SCP while only VD was affected in DCP is striking.

Clinically, SLE-associated retinopathy predominantly presents as a disease affecting the retinal vessels, often affecting the retinal arteries and, rarely, the veins 7 . Consequently, in OCTA imaging, one would expect the superficial capillary plexus (SCP), which is anatomically part of the retinal arteriolar system 15 , to be more affected than the deep capillary plexus (DCP), which lies closer to the retinal veins. Therefore, the changes observed in this study may reflect subclinical retinal vasculitis. It is also possible that other subclinical vascular alterations, possibly associated with SLE, such as subclinical atherosclerosis are observed. Such atherosclerotic changes, which can also be observed in other organs 16 , 17 , predominantly affect arteries and arterioles, which would also explain the predominant affection of the SCP.

Twenty-One out of 25 patients in the observed SLE-cohort were treated using Plaquenil. While there were no signs of Plaquenil-related retinal toxicity, there are is some evidence that prolonged Plaquenil exposure might lead to lower VD 18 , which could also influence the findings of this study. As the percentage of SLE patients taking Plaquenil in our cohort was high (84%), and the overall cohort was quite small, our data does not allow any statements on whether Plaquenil intake had a significant effect on VD in this study cohort.

Limitations of this study include its retrospective nature, small sample size, a skew towards female patients in the SLE group and a difference in age in between the SLE group and the healthy cohort. A significant limitation is the high percentage of patients taking Plaquenil as treatment, which, combined with the small sample size, might be a significant confounder. This warrants additional exploration in a larger cohort, ideally with more patients not taking Plaquenil. The study design, being cross-sectional, does not allow to infer prognostic value or clinical significance of the findings discussed above. This should be investigated further by larger, ideally prospective cohort studies.

Further research is warranted to establish whether OCTA imaging can be a tool to facilitate diagnosing early SLE and monitoring disease activity.

## References

[1] AzevedoL GBBiancardiA LSilvaR ALupus retinopathy: epidemiology and risk factorsArq Bras Oftalmol20218439540110.5935/0004-2749.2021007634287516 PMC11826623

[2] DammaccoRSystemic lupus erythematodes and ocular involvement: an overviewClin Exp Med20181813514929243035 10.1007/s10238-017-0479-9

[3] Hochberg M C Updating the American college of rheumatology revised criteria for the classification of systemic lupus erythematodes Arthritis Rheum 1997 40 1725 1725 10.1002/art.1780400928 9324032

[4] TanE MCohenA SFriesJ FThe 1982 revised criteria for the classification of systemic lupus erythematodesArthritis Rheum198225127112777138600 10.1002/art.1780251101

[5] ReadRClinical mini-review: systemic lupus erythematodes and the eyeOcul Immunol Inflamm200412879915512979 10.1080/09273940490895308

[6] PapagiannuliERhodesBWallaceG RSystemic lupus erythematodes: An update for ophthalmologistsSurv Ophthalmol201661658226197421 10.1016/j.survophthal.2015.06.003

[7] DaviesJ BRaoP KOcular manifestations of systemic lupus erythematodesCurr Opin Ophthalmol20081951251818998618 10.1097/icu.0b013e3283126d34

[8] ConigliaroPTriggianesePDraghessiGEvidence for the Detection of Subclinical Retinal Involvement in Systemic Lupus Erythematodes and Sjögren Syndrome: A Potential Association with TherapiesInt Arch Allergy Immunol2018177455629902805 10.1159/000488950

[9] SpaideR FKlancnikJ MCooneyM JRetinal Vascular Layers Imaged by Fluorescein Angiography and Optical Coherence Tomography AngiographyJAMA Ophthalmol20151334525317632 10.1001/jamaophthalmol.2014.3616

[10] WiestM RJToroM DNowakAGlobotrioasylsphingosine Levels and Optical Coherence Tomography Angiography in Fabry Disease PatientsJ Clin Med202110109333807900 10.3390/jcm10051093PMC7961664

[11] HsiaYChangH LWangT HThe Artifacts in Macular and Peripapillary OCT Angiography in Patients with Different Severities of GlaucomaOphthalmol Sci2026610096441377060 10.1016/j.xops.2025.100964PMC12686908

[12] KojimaKIchinoseKUmedaMEnhancing systemic lupus erythematodes treatment outcomes with an early initiation of belimumab: insights from a multicenter retrospective study within the first five yearsArthritis Res Ther20252711640442811 10.1186/s13075-025-03581-0PMC12121007

[13] Basiony A I Elgouhary S M Mohamed H E Assessment of retinal microvascular changes in patients with systemic lupus erythematodes using optical coherence tomography angiography Int J Retina Vitr 2025 11 55 10.1186/s40942-025-00677-2 PMC12060311 40340989

[14] ConigliaroPGianniniCFerrignoSAssessment of microvascular involvement in lupus nephritis patients by retinal OCT-angiography and kidney biopsiesClin Exp Rheumatol20234158158835916306 10.55563/clinexprheumatol/p1q482

[15] Garrity S T Iafe N A Phasukkijwatana N Quantitative Analysis of Three Distinct Retinal Capillary Plexuses in Healthy Eyes Using Optical Coherence Tomography Angiography Invest Opthalmol Vis Sci 2017 58 5548 5555 10.1167/iovs.17-22036 29075766

[16] WangPMaoY MZhaoC NIncreased Pulse Wave Velocity in Systemic Lupus Erythematodes: A Meta-AnalysisAngiology20186922823528635303 10.1177/0003319717715964

[17] KoletsosNLazaridisATriantafyllouAAccumulation of Microvascular Target Organ Damage in Systemic Lupus Erythematodes Patients Is Associated with Increased Cardiovascular RiskJ Clin Med202413214038610905 10.3390/jcm13072140PMC11012611

[18] VasilijevićJ BKovačevićI MDijanaRDačićBMarićGStanojlovićSOptical coherence tomography angiography parameters in patients taking hydroxychloroquine therapyIndian J Ophthalmol2023713399340537787242 10.4103/IJO.IJO_740_23PMC10683689

